# Global pharmacovigilance reporting: comparative analysis of adverse event obligations across five major regulatory authorities

**DOI:** 10.3389/fdsfr.2026.1821686

**Published:** 2026-05-07

**Authors:** Carlos G. Bernaus, Maria Lilia Bournissen

**Affiliations:** 1 University of Business and Social Sciences, Buenos Aires, Argentina; 2 Independent Researcher, Boston, United States

**Keywords:** adverse event reporting, aggregate safety analysis, development safety update report, health authorities, individual case safety reports, international drug safety, pharmacovigilance

## Abstract

**Background:**

As pharmaceutical development spans multiple continents, marketing authorization holders must simultaneously comply with distinct adverse event reporting frameworks of major health authorities. Although International Council for Harmonisation guidelines provide shared foundations, meaningful differences persist in reporting timelines, case thresholds, and special-situation obligations across jurisdictions.

**Methods:**

A narrative review of primary regulatory legislation, guidance documents, peer-reviewed literature, and International Council for Harmonisation guidelines was conducted for five major regulatory authorities: Health Canada, the U.S. Food and Drug Administration, the European Medicines Agency, Japan’s Pharmaceuticals and Medical Devices Agency, and China’s National Medical Products Administration. These authorities collectively represent the majority of global pharmaceutical market value and clinical trial activity, and each is either an International Council for Harmonisation founding or observer member. Comparative summary tables and decision algorithms were synthesized from authoritative sources across clinical trial, post-marketing, medical device, vaccine, and special-population reporting contexts.

**Results:**

All five authorities share International Council for Harmonisation seriousness criteria and require expedited seven-calendar-day reporting for fatal or life-threatening suspected unexpected serious adverse reactions in clinical trials. Key jurisdictional differences include: China’s unique 24-h obligation for post-marketing deaths; the European Medicines Agency’s device vigilance timelines (10 calendar days for deaths, two calendar days for serious public health threats); the Food and Drug Administration’s 30-calendar-day default for manufacturer device reports; the European Medicines Agency’s 90-calendar-day obligation for non-serious individual case safety reports from the European Economic Area; and Japan’s 30-calendar-day timeline for serious expected domestic adverse drug reactions. The Food and Drug Administration’s December 2025 final guidance on sponsor responsibilities further consolidated aggregate analysis obligations, and the proposed transition from the Investigational New Drug Annual Report to the Development Safety Update Report format signals growing emphasis on periodic safety assessment.

**Conclusion:**

Effective pharmacovigilance depends on the interplay between expedited individual case reporting and aggregate periodic assessments, including Development Safety Update Reports and Periodic Benefit-Risk Evaluation Reports. A structured decision-tree approach enables marketing authorization holders to manage multi-authority reporting obligations systematically, while ongoing regulatory convergence—exemplified by global adoption of the Development Safety Update Report—reduces submission format burdens.

## Introduction

The post-thalidomide era transformed medicines regulation globally. The tragedy of the early 1960s catalyzed the establishment of national pharmacovigilance systems and ultimately the World Health Organization Programme for International Drug Monitoring, inaugurated in 1968 (World Health Organization, 2002). Since then, the scientific and regulatory architecture of drug safety has expanded dramatically, encompassing spontaneous reporting systems, active surveillance databases, risk management plans, and periodic safety update reports. Today, marketing authorization holders operating across multiple markets face a complex patchwork of reporting obligations.

The International Council for Harmonisation of Technical Requirements for Pharmaceuticals for Human Use—a joint initiative of regulatory authorities and the pharmaceutical industry from Europe, Japan, and the United States—has produced a suite of harmonized guidelines, including E2A (clinical safety data management: definitions and standards for expedited reporting), E2B(R3) (electronic transmission of individual case safety reports), E2C(R2) (periodic benefit-risk evaluation reports), E2D (post-approval safety data management), E2E (pharmacovigilance planning), and E2F (development safety update reports) (International Council for Harmonisation, 1994; International Council for Harmonisation, 2016a; International Council for Harmonisation, 2012; International Council for Harmonisation, 2003; International Council for Harmonisation, 2004; International Council for Harmonisation, 2010). Despite this framework, meaningful regulatory divergence persists.

From a patient safety standpoint, the ultimate purpose of pharmacovigilance reporting is to enable rapid signal detection and risk communication. Failures in reporting—whether through delayed submission, misclassification of seriousness, or misdirection to the wrong authority—directly compromise this goal and may constitute regulatory infractions. Conversely, the administrative burden of multi-authority compliance diverts pharmacovigilance resources from substantive safety science. Importantly, the pharmacovigilance system relies on a complementary architecture: expedited individual case reporting enables rapid detection of new or unusually severe safety signals, while aggregate periodic assessments—Development Safety Update Reports during clinical development and Periodic Benefit-Risk Evaluation Reports post-marketing—provide the structured framework for comprehensive, contextual evaluation of a product’s evolving safety profile (International Council for Harmonisation, 2012; International Council for Harmonisation, 2010). Recognizing and leveraging this interplay is essential for effective safety oversight.

This study provides a structured comparative analysis of reporting obligations across five major regulatory authorities most commonly encountered in global clinical development and post-marketing surveillance programs: Health Canada, the U.S. Food and Drug Administration, the European Medicines Agency, the Pharmaceuticals and Medical Devices Agency of Japan, and China’s National Medical Products Administration. Together, these five authorities represent the majority of global pharmaceutical market value and a growing proportion of clinical trial activity. Our objective is to identify areas of harmonization and divergence, evaluate each framework in the context of the broader pharmacovigilance ecosystem—including aggregate reporting—and provide practical guidance for marketing authorization holders managing multi-jurisdictional compliance obligations.

## Materials and methods

### Rationale for selection of regulatory authorities

The five authorities selected for this analysis—Health Canada, the U.S. Food and Drug Administration, the European Medicines Agency, the Pharmaceuticals and Medical Devices Agency of Japan, and China’s National Medical Products Administration—were chosen based on four criteria. First, they collectively represent the largest pharmaceutical markets by value and regulatory significance. Second, they include all three founding International Council for Harmonisation regions (European Union, Japan, United States) plus two major non-founding markets (Canada as an International Council for Harmonisation observer member, and China as the world’s second-largest pharmaceutical market). Third, each authority administers pharmacovigilance obligations for large patient populations, providing diverse perspectives on reporting infrastructure scalability. Fourth, products seeking global registration typically require submissions to most or all of these authorities, making their combined requirements the practical standard for multinational marketing authorization holders.

Several additional authorities merit consideration but were excluded to maintain analytical depth. The United Kingdom’s Medicines and Healthcare products Regulatory Agency, which operates independently since Brexit, applies a framework substantively similar to the European Medicines Agency’s pre-exit requirements and continues to accept International Council for Harmonisation E2B(R3) formatted submissions. The Therapeutic Goods Administration of Australia, another International Council for Harmonisation observer member, employs reporting obligations broadly aligned with the European Medicines Agency model. India’s Pharmacovigilance Programme, administered by the Indian Pharmacopoeia Commission, oversees the world’s second-largest population but remains in a developmental phase with respect to spontaneous reporting infrastructure and marketing authorization holder obligations (Kalaiselvan et al., 2016). Future comparative analyses incorporating these and other authorities would further enhance global understanding of pharmacovigilance divergence.

### Literature search strategy

We conducted a comprehensive narrative review of primary regulatory legislation, guidance documents, International Council for Harmonisation guidelines, and peer-reviewed literature governing adverse event reporting to the five selected regulatory authorities. The review was conducted between January 2024 and December 2025, with literature searches updated through December 2025.

### Data sources and inclusion criteria

Primary sources included current versions of regulatory legislation and guidance documents as of December 2025. We systematically identified and reviewed the complete suite of International Council for Harmonisation guidelines relevant to pharmacovigilance, including E2A (clinical safety data management and expedited reporting definitions), E2B(R3) (electronic transmission of individual case safety reports), E2C(R2) (periodic benefit-risk evaluation reports), E2D (post-approval safety data management), E2E (pharmacovigilance planning), E2F (development safety update reports), and E6(R2) (good clinical practice) (International Council for Harmonisation, 1994; International Council for Harmonisation, 2016a; International Council for Harmonisation, 2012; International Council for Harmonisation, 2003; International Council for Harmonisation, 2004; International Council for Harmonisation, 2010; International Council for Harmonisation, 2016b).

For Health Canada, we reviewed the Food and Drugs Act and its subordinate Food and Drug Regulations with particular focus on Divisions C.01 (marketed drugs), C.05 (clinical trials), and C.08 (new drugs and marketing authorizations), along with the Medical Devices Regulations and guidance documents from the Marketed Health Products Directorate (Government of Canada, 2023a; Government of Canada, 2023b; Government of Canada, 2023c; Health Canada, 2019). The U.S. Food and Drug Administration framework was examined through the Federal Food, Drug, and Cosmetic Act and implementing regulations in Title 21 Code of Federal Regulations Parts 310 (new drugs), 312 (investigational new drug applications), 314 (new drug applications), 600 (biological products), and 803 (medical device reporting), supplemented by relevant FDA guidance documents including the December 2025 final guidances on sponsor and investigator responsibilities for Investigational New Drug safety reporting and the proposed rule on transitioning Investigational New Drug annual reporting to the Development Safety Update Report format (U.S. Food and Drug Administration, 2023; U.S. Government Publishing Office, 2023a; U.S. Government Publishing Office, 2023b; U.S. Government Publishing Office, 2023c; U.S. Government Publishing Office, 2023d; U.S. Government Publishing Office, 2023e; U.S. Food and Drug Administration, 2012; U.S. Food and Drug Administration, 2025a; U.S. Food and Drug Administration, 2025b; U.S. Food and Drug Administration, 2022).

The European Medicines Agency’s regulatory framework was analyzed through Regulation (EC) No 726/2004, Directive 2001/83/EC as amended by Directive 2010/84/EU, Commission Implementing Regulation (EU) No 520/2012, the complete Good Pharmacovigilance Practices module series (Modules I through XVI), Medical Device Regulation (EU) 2017/745, and Clinical Trials Regulation (EU) No 536/2014 (European Parliament and Council, 2004; European Parliament and Council, 2001; European Parliament and Council, 2010; European Commission, 2012; European Medicines Agency, 2023; European Parliament and Council, 2017; European Parliament and Council, 2014). For Japan, we examined the Pharmaceutical and Medical Device Act, ministerial ordinances on Good Vigilance Practice and Good Post-Marketing Study Practice, and Pharmaceuticals and Medical Devices Agency guidance documents (Ministry of Health et al., 2021a; Ministry of Health et al., 2021b; Ministry of Health et al., 2021c; Pharmaceuticals and Medical Devices Agency, 2022). China’s framework was evaluated through the Drug Administration Law (2019), Pharmacovigilance Quality Management Regulations (2021), Vaccine Administration Law (2019), and National Medical Products Administration guidance documents (National People’s Congress, 2019a; National Medical Products Administration, 2021; National People’s Congress, 2019b; National Medical Products Administration, 2022).

In addition to government and regulatory sources, we searched PubMed and Embase databases using combinations of terms including “pharmacovigilance,” “adverse event reporting,” “safety reporting regulations,” “regulatory comparison,” and the names of the five regulatory authorities. We identified peer-reviewed articles addressing pharmacovigilance systems in developing countries, signal detection methodology, and assessments of spontaneous reporting systems. Key peer-reviewed sources informing our analysis include Pacurariu et al. (2014) on signal detection in EudraVigilance, Koutkias and Jaulent (2015) on computational pharmacovigilance approaches, Pal et al. (2013) on the WHO Programme for International Drug Monitoring, Maigetter et al. (2015) on comparative pharmacovigilance legislation, and a recent narrative review by Khan et al. (2023) on pharmacovigilance in high-income countries. Inclusion criteria required that sources be directly relevant to adverse event reporting obligations, publicly accessible, and available in English or with verified English translations for non-English sources.

### Regulatory framework analysis

For each authority, we systematically extracted and analyzed the legislative basis for pharmacovigilance obligations, reporting timelines for serious and non-serious adverse events categorized by fatal/life-threatening events, other serious unexpected events, serious expected events, non-serious events, device-related incidents, and special situations. We documented authority-specific interpretations of seriousness criteria, expectedness determination, case validity requirements, and reference safety information definitions. Electronic reporting platforms, data formats, and technical requirements were catalogued for each system. Special requirements were analyzed across clinical trial safety reporting, post-marketing surveillance, medical device vigilance, vaccine adverse events, pregnancy exposures and pediatric populations, and situations involving lack of efficacy, medication errors, and abuse or misuse. Finally, we examined requirements for aggregate safety reports including Development Safety Update Reports, Periodic Safety Update Reports, and Periodic Benefit-Risk Evaluation Reports.

### Comparative assessment methodology

We developed a structured comparative framework evaluating each authority across five dimensions of safety signal collection effectiveness. The scope of data collection dimension assessed breadth of reportable events (serious *versus* non-serious, expected *versus* unexpected), geographic coverage requirements (domestic *versus* foreign cases), special population and special situation provisions, and integration of multiple data sources. Timeline realism and data quality evaluation considered the practicality of reporting deadlines given operational workflows, balance between urgency and completeness of initial reports, provisions for follow-up information, and alignment with International Council for Harmonisation standards.

Infrastructure and signal detection capability assessment examined centralization *versus* fragmentation of reporting systems, electronic submission capabilities and data standards, public access to safety databases, analytical tools and signal detection methodologies, and statistical power based on population coverage. Special population and situation coverage analysis focused on pregnancy exposure and congenital anomaly reporting, pediatric adverse event requirements, lack of efficacy provisions, medication error and use error reporting, and handling of abuse, misuse, and occupational exposure. Operational feasibility evaluation considered administrative burden on marketing authorization holders, clarity and consistency of requirements, harmonization with international standards, language and translation requirements, and duplicate reporting obligations.

For each dimension, we assigned qualitative assessments (strong, moderate, or limited) based on the presence and characteristics of specific regulatory provisions. These assessments represent the authors’ informed judgments based on the regulatory evidence reviewed and should be interpreted as such rather than as empirically validated rankings.

### Decision algorithm development

Based on the extracted regulatory requirements, we developed decision algorithms to enable systematic case triage from initial receipt to reporting obligation determination. These algorithms incorporate case validity assessment using the four minimum elements per International Council for Harmonisation E2B(R3) (identifiable reporter, identifiable patient, suspect medicinal product, and suspected adverse reaction), seriousness determination using International Council for Harmonisation E2A criteria, expectedness assessment through comparison to reference safety information, authority-specific timeline calculation, special situation identification, and destination system routing. The algorithms were designed to accommodate the most stringent applicable requirements when multiple authorities have jurisdiction over a single case, enabling marketing authorization holders to implement global workflows that ensure compliance across all five regulatory frameworks. The complete decision algorithm is available as Supplementary Material.

## Results

### Regulatory frameworks overview

All five authorities have established comprehensive pharmacovigilance frameworks anchored in primary legislation and implemented through subordinate regulations and guidance documents. Table 1 summarizes the legislative basis and primary reporting systems for each authority.

**TABLE 1:** Comparative summary of key reporting parameters across five regulatory authorities.

Parameter	Health Canada^3^	FDA (US)	EMA/EU NCAs (EU)^1^	PMDA (Japan)	NMPA (China)
SUSAR — fatal / life-threatening (clinical trials)	7 calendar days	7 calendar days	7 calendar days	7 calendar days	7 calendar days
SUSAR — other serious unexpected (clinical trials)	15 calendar days	15 calendar days	15 calendar days	15 calendar days	15 calendar days
Post-market serious unexpected ADR	15 calendar days^2^	15 calendar days	15 calendar days	15 calendar days	15 calendar days
Post-market serious expected ADR	15 calendar days (domestic)^2^	Periodic reports (PADER / PBRER / DSUR)	15 calendar days (EEA)	15 calendar days (domestic fatal, listed) 30 calendar days (other domestic serious listed; foreign fatal listed)	15 calendar days
Overseas suspension / withdrawal or major drug-quality safety event	Notification to Regulatory Operations and Enforcement Branch; no single statutory clock	DSCSA illegitimate-product notification: 24 hours (Form FDA 3911)	Notification “without undue delay” to NCAs and EMA; Rapid Alert transmitted “without any delay” if serious risk to public health	Notification to MHLW under PMD Act Art. 68-11 (recall classification timelines)	MAH immediate control and notification to provincial authority; provincial authority reports to NMPA within 24 hours
Non-serious ICSR	Not required individually	Not required individually (captured in PADER)	90 calendar days (EEA cases)	Not required individually	Not required individually
Device incident — death or serious deterioration in health	10 calendar days (manufacturer / importer)	30 calendar days (manufacturer baseline); 5 work days (remedial action or FDA-requested); 10 work days (user facilities)	10 calendar days (manufacturer to NCA, EU MDR Art. 87(5))	15 calendar days (AE15: domestic death or unlisted serious)	24 hours (overseas device adverse events to NMPA)
Lack of efficacy provision	15 calendar days for unusual failure in efficacy of new drugs (domestic)	Reported only if seriousness criteria met	Reported only if seriousness criteria met	Not defined as a standalone reportable category; reported as ADR when seriousness and unexpectedness criteria are met	Reported when events meet seriousness criteria per GVP (2021); heightened surveillance under antimicrobial stewardship policies
Reference documents for expectedness assessment	Investigator's Brochure (IB) (CT) Canadian Product Monograph (PM)	Investigator's Brochure (IB) (CT) Prescribing Information (USPI / package insert) (PM)	Reference Safety Information defined section of IB, or SmPC Section 4.8 (CT) SmPC Section 4.8 (PM)	Investigator's Brochure (CT) Japanese Package Insert Article 52, PMD Act (PM)	Investigator's Brochure (CT) Chinese Package Insert (PM)
Annual aggregate report	Annual summary; PSUR / PBRER	PADER; IND Annual Report (transitioning to DSUR)	DSUR (development); PSUR / PBRER (post-market)	DSUR; re-examination report	DSUR; periodic safety report; Pharmacovigilance Annual Summary Report
E2B(R3) adoption status	E2B(R2) remains in operational use for Canada Vigilance Program E2B(R3) not yet mandated	Acceptance from 16 January 2024 (post-market) and 1 April 2024 (pre-market / IND) Mandatory via ESG NextGen from 1 October 2026 (91 FR 17284)	Since 22 November 2017 Mandatory ISO / ICH E2B(R3) from 30 June 2022	Transitional acceptance from April 2016 Mandatory from 1 April 2019	IND SUSARs: mandatory from May 2019 (CDE) Post-market ADRs: mandatory from 1 July 2022 (CDR / NMPA)
Language requirement	English or French	English	English accepted in EudraVigilance; NCA submissions in local official language	Japanese commonly required for domestic reports	Chinese mandatory for domestic reports; international ICSRs may include English narrative
Counterfeit / falsified / adulterated product reporting	Any ADR from a suspected counterfeit: C.01.017 (15 days, serious unexpected) Suspected cases reported to Regulatory Operations and Enforcement Branch	Field Alert Report: 3 working days (NDA / ANDA holders) per 21 CFR 314.81(b)(1) DSCSA illegitimate-product notification: 24 hours (Form FDA 3911)	Notification to EMA and concerned NCAs “without undue delay” (FMD 2011/62/EU; Del. Reg. (EU) 2016/161 Art. 37) Rapid Alert to “without any delay” where serious risk to public health (Dir. 2001/83/EC Art. 117a(3))	Recall notification to MHLW / PMDA under PMD Act Art. 68-11 Timeline classified by recall risk class (Class I–III)	MAH: immediate awareness (Drug Manufacturing Provisions Art. 65) Provincial authority: reports to NMPA within 24 hours Drug Administration Law Arts. 98, 116 define counterfeit and inferior drugs

Comparative framework for individual case safety report (ICSR) and quality-related reporting obligations across Health Canada (Canada), the Food and Drug Administration (United States), the European Medicines Agency together with national competent authorities (European Union / European Economic Area), the Pharmaceuticals and Medical Devices Agency (Japan), and the National Medical Products Administration (China). Timelines refer to marketing authorization holder (MAH) or applicant obligations unless otherwise indicated. Unless explicitly stated, all timelines denote calendar days measured from the date the MAH / manufacturer first becomes aware of the information (Day 0). Medical-device vigilance in the European Union is governed by Regulation (EU) 2017/745 and coordinated by national competent authorities under the Medical Device Coordination Group (MDCG), not by the EMA.

Abbreviations: ADR, adverse drug reaction; CDE, center for drug evaluation (NMPA); CDR, center for drug reevaluation (NMPA); DSCSA, drug supply chain security act (United States); CT, clinical trial cases; DSUR, development safety update report; EEA, european economic area; ESG, electronic submissions gateway (FDA); FAR, field alert report; FMD, falsified medicines directive (Directive 2011/62/EU); GVP, good pharmacovigilance practice; ICH, international council for harmonisation of technical requirements for pharmaceuticals for human use; ICSR, individual case safety report; IND, investigational new drug application; MAH, marketing authorisation holder; MDR, medical device regulation (EU) 2017/745; MDCG, medical device coordination group; MHLW, ministry of health, labour and welfare (Japan); NCA, national competent authority; NDA / ANDA, new drug application / Abbreviated New Drug Application; PADER, periodic adverse drug experience report; PBRER, periodic benefit–risk evaluation report; PMD Act, act on securing quality, efficacy and safety of pharmaceuticals, medical devices, regenerative and cellular therapy products, gene therapy products and cosmetics (Japan); PM, post-marketing products; PSUR, periodic safety update report; SUSAR, suspected unexpected serious adverse reaction.

1 Medical-device vigilance in the European Union is governed by Regulation (EU) 2017/745 (MDR) and coordinated by national competent authorities under the Medical Device Coordination Group (MDCG), not by the European Medicines Agency. For medicinal products, EMA operates EudraVigilance on behalf of the EU medicines regulatory network.

2 Solicited post-market serious unlikely cases are reportable to Health Canada.

3 An identifiable patient is one whom a case processor can reasonably verify as a real individual (rather than hypothetical) and distinguish from other patients for de-duplication purposes. Consequently, a bare reference to ‘a patient’ is generally treated as insufficient under ICH E2D criteria; Health Canada’s guidance, however, explicitly accepts this formulation as meeting the identifiability threshold.

### Health Canada

Health Canada’s pharmacovigilance framework is anchored in the Food and Drugs Act and its subordinate Food and Drug Regulations, particularly Division C.01 for marketed drugs, Division C.05 for clinical trials, and Division C.08 for new drugs and marketing authorizations (Government of Canada, 2023a; Government of Canada, 2023b). The Canada Vigilance Programme, operated by the Marketed Health Products Directorate, receives individual case safety reports submitted by marketing authorization holders electronically in International Council for Harmonisation E2B(R2,R3) format through the Canada Vigilance gateway (Health Canada, 2019). Public- and healthcare professional-facing channels (MedEffect and online forms) supplement but do not replace marketing authorization holder electronic submissions.

Two provisions of Division C.01 warrant specific attention. First, C.01.017(2)(b) requires expedited reporting within 15 calendar days of an unusual failure of a drug to produce its intended effect for domestic (Canadian) cases (Government of Canada, 2023b). This operates independently of International Council for Harmonisation seriousness criteria and captures unexpected efficacy patterns that may signal product quality issues or incorrect dosing guidance, particularly for life-sustaining therapies. Second, C.01.018 mandates that manufacturers prepare an annual summary report of all adverse drug reactions and serious adverse drug reactions received or identified during the previous 12-month period, serving as a structured review mechanism distinct from expedited case reporting and Periodic Safety Update Reports (Government of Canada, 2023b).

For medical devices, the Medical Devices Regulations impose separate incident-reporting obligations administered by the Medical Devices Bureau, including a 10-calendar-day initial report for incidents that have led to death (Government of Canada, 2023c).

### U.S. food and drug administration

The Food and Drug Administration’s pharmacovigilance obligations derive principally from the Federal Food, Drug, and Cosmetic Act and implementing regulations in Title 21 Code of Federal Regulations Parts 310, 312, 314, 600, and 803 (U.S. Food and Drug Administration, 2023; U.S. Government Publishing Office, 2023a; U.S. Government Publishing Office, 2023b; U.S. Government Publishing Office, 2023c; U.S. Government Publishing Office, 2023d; U.S. Government Publishing Office, 2023e). The FDA Adverse Event Reporting System receives post-marketing individual case safety reports submitted electronically through the Electronic Submissions Gateway (U.S. Food and Drug Administration, 2012). Safety reporting during clinical trials is governed by the Investigational New Drug Safety Reporting regulations at 21 Code of Federal Regulations 312.32, substantially revised in 2010 to emphasize aggregate analysis alongside individual case reporting (U.S. Government Publishing Office, 2023b).

In December 2025, the Food and Drug Administration issued two final guidances that replaced earlier guidance documents on Investigational New Drug safety reporting: “Sponsor Responsibilities—Safety Reporting Requirements and Safety Assessment for IND and Bioavailability/Bioequivalence Studies” and “Investigator Responsibilities—Safety Reporting for Investigational Drugs and Devices” (U.S. Food and Drug Administration, 2025a; U.S. Food and Drug Administration, 2025b). The sponsor guidance consolidates and updates recommendations for compliance with 21 Code of Federal Regulations 312.32. A central principle is that the sponsor must adopt a systematic approach to safety surveillance across the entire development program, including a process for promptly reviewing, evaluating, and managing accumulating data on all serious adverse events and related safety information (U.S. Food and Drug Administration, 2025a). The guidance clarifies that the sponsor—not individual investigators—is best positioned to determine whether there is a reasonable possibility that the study drug caused an adverse event, thereby establishing whether the event qualifies as a suspected adverse reaction warranting an Investigational New Drug safety report. The Food and Drug Administration emphasizes that few events can be judged to be drug-related on the basis of one or a small number of occurrences, and that indiscriminate reporting of all individual serious adverse events without aggregate assessment can obscure important safety information (U.S. Food and Drug Administration, 2025a). Accordingly, sponsors should assess all accumulating data at regular intervals to make decisions about whether information meets the criteria for expedited reporting, to update reference safety information in the Investigator Brochure, and to identify new safety signals. This aggregate analysis framework recognizes that individual case reports are most informative when evaluated within the context of the developing safety profile, including medical review of individual cases, medical monitoring of individual studies, and cumulative assessment across the program (U.S. Food and Drug Administration, 2025a).

Separately, the Food and Drug Administration has proposed rulemaking to replace the Investigational New Drug Annual Report (21 Code of Federal Regulations 312.33) with a Development Safety Update Report format consistent with International Council for Harmonisation E2F (U.S. Food and Drug Administration, 2022). The proposed FDA Development Safety Update Report would require more comprehensive content than the current annual report, including global clinical data, cumulative subject exposure, interval and cumulative safety data, a listing of all known important risks, and integrated safety assessments. This proposal signals the Food and Drug Administration’s alignment with the global trend toward the Development Safety Update Report as the standard for annual safety assessment during clinical development.

Medical device reporting operates under the Medical Device Reporting regulation at 21 Code of Federal Regulations Part 803, with manufacturer submissions generally due in 30 calendar days; a five-working-day report applies only when remedial action is necessary to prevent unreasonable risk of substantial harm to the public or when specifically requested by the Food and Drug Administration (U.S. Government Publishing Office, 2023e). Vaccine post-marketing adverse experience reporting by manufacturers follows 21 Code of Federal Regulations 600.80 (15-day “Alert reports” for serious and unexpected events); the Vaccine Adverse Event Reporting System functions as the reporting system jointly managed by the Food and Drug Administration and Centers for Disease Control and Prevention (U.S. Government Publishing Office, 2023d).

### European medicines agency

The European Medicines Agency’s pharmacovigilance framework reflects the multi-state structure of the European Union. The primary legislative instruments are Regulation (EC) No 726/2004 and Directive 2001/83/EC, substantially amended by Directive 2010/84/EU and Commission Implementing Regulation (EU) No 520/2012 (European Parliament and Council, 2004; European Parliament and Council, 2001; European Parliament and Council, 2010; European Commission, 2012). Good Pharmacovigilance Practices Modules I through XVI provide detailed operational guidance (European Medicines Agency, 2023). Individual case safety reports for both centrally authorized products and nationally authorized products are submitted by marketing authorization holders directly to EudraVigilance in E2B(R3) format under the “single submission” model introduced in 2017 (European Medicines Agency, 2023).

Medical device vigilance operates under the Medical Device Regulation (EU) 2017/745, administered nationally through competent authorities and centrally through EUDAMED (European Parliament and Council, 2017). Under Medical Device Regulation Article 87, timelines are two calendar days for a serious public health threat, 10 calendar days for incidents that led to death or unanticipated serious deterioration in health, and 15 calendar days for other serious incidents (European Parliament and Council, 2017).

### Pharmaceuticals and medical devices agency (Japan)

Japan’s pharmacovigilance system is governed by the Pharmaceutical and Medical Device Act and ministerial ordinances on Good Vigilance Practice and Good Post-Marketing Study Practice (Ministry of Health et al., 2021a; Ministry of Health et al., 2021b; Ministry of Health et al., 2021c). The Pharmaceuticals and Medical Devices Agency conducts pharmacovigilance on behalf of the Ministry of Health, Labour and Welfare and maintains a centralized safety database (Pharmaceuticals and Medical Devices Agency, 2022). A distinctive feature of Japan’s system is the re-examination period—typically four to 10 years post-approval—during which marketing authorization holders are subject to enhanced post-marketing surveillance obligations under Good Post-Marketing Study Practice, including intensive case collection and periodic re-examination reports (Ministry of Health et al., 2021c).

A critical operational aspect of Japan’s system is the case acceptance workflow for foreign cases. The Pharmaceuticals and Medical Devices Agency’s reporting timelines (Day 0) are triggered upon the marketing authorization holder’s formal acceptance of a case into their pharmacovigilance system—after validation of minimum case elements (identifiable patient, suspect product, adverse reaction, identifiable reporter)—rather than the moment of initial awareness (Pharmaceuticals and Medical Devices Agency, 2022). Japan has progressively adopted International Council for Harmonisation guidelines; E2B(R3) is accepted for electronic individual case safety report submission, although Japanese-language narratives are often expected or required for domestic cases (Pharmaceuticals and Medical Devices Agency, 2022).

### National medical products administration (China)

China’s pharmacovigilance system has undergone substantial reform since the 2019 Drug Administration Law and the 2021 Pharmacovigilance Quality Management Regulations (National People’s Congress, 2019a; National Medical Products Administration, 2021). These reforms introduced a marketing authorization holder-centered model and closer alignment with international standards. The National Centre for Adverse Drug Reaction Monitoring administers the national adverse drug reaction reporting database (National Medical Products Administration, 2022). Reports are submitted through provincial adverse drug reaction monitoring centers and the national system. International Council for Harmonisation E2B(R3) is being implemented progressively; Chinese-language reporting remains mandatory for domestic cases (National Medical Products Administration, 2022). A defining characteristic is the 24-h preliminary reporting obligation for post-marketing deaths, followed by a complete individual case safety report within 15 calendar days (National Medical Products Administration, 2021).

### Seriousness, expectedness, and case validity

Seriousness is the primary triage criterion in all five systems. International Council for Harmonisation E2A defines a serious adverse event as one that results in death, is life-threatening, requires inpatient hospitalization or prolongation thereof, results in persistent or significant disability or incapacity, is a congenital anomaly or birth defect, or constitutes an important medical event (International Council for Harmonisation, 1994). This definition has been adopted substantially verbatim by all five authorities.

Expectedness—whether an adverse reaction is listed in the current reference safety information—determines the reporting category and, in clinical trials, whether a case is a suspected unexpected serious adverse reaction. During development, the reference safety information is the current Investigator Brochure; post-marketing, it is the locally approved product information (Product Monograph in Canada, Prescribing Information in the United States, Summary of Product Characteristics in the European Union, Package Insert in Japan, and the Chinese approved label). A serious adverse drug reaction that is listed is generally not subject to expedited reporting; jurisdictional exceptions include Health Canada and the European Medicines Agency, which require 15-day expedited reporting for serious expected domestic cases.

Before any reporting obligation is assessed, the case must satisfy the minimum validity criteria. Per International Council for Harmonisation E2B(R3), a valid individual case safety report requires four elements: an identifiable reporter, an identifiable patient, a suspect medicinal product, and a suspected adverse reaction (International Council for Harmonisation, 2016a). Cases lacking any of these elements should be pursued for follow-up and should not be submitted until all four are present; once validity is established, submission should not be delayed to obtain additional details.

In the context of clinical trial safety reporting, particularly under the Food and Drug Administration’s Investigational New Drug framework, an adverse event must meet three criteria before it is reported as an Investigational New Drug safety report: it must be serious, it must be unexpected (not listed in the current Investigator Brochure), and there must be a reasonable possibility that the study drug caused the event—that is, it must qualify as a suspected adverse reaction (International Council for Harmonisation, 1994; U.S. Food and Drug Administration, 2025a). All three criteria must be satisfied; if any one is not met, the event should not be submitted as an expedited safety report. Determining the third criterion—causal association—has historically been the most challenging, requiring clinical judgment informed by the totality of available evidence. The December 2025 Food and Drug Administration final guidance on sponsor responsibilities acknowledges the difficulty of these determinations and states that the Agency will focus primarily on the robustness of the sponsor’s process and the reasoning underlying the sponsor’s decision, rather than second-guessing individual causality assessments (U.S. Food and Drug Administration, 2025a). This emphasis on process robustness and documented justification provides an opportunity to leverage the expertise of multidisciplinary safety management teams conducting regular program-level reviews of accumulating safety data, which is vital not only for early signal detection but also for generating the context needed to reduce false-positive safety reports. Although all five authorities require causality assessment for expedited reporting, the Food and Drug Administration’s 2025 guidance provides the most detailed articulation of the sponsor’s role in this determination.

### Clinical trial safety reporting requirements

The regulatory instruments governing clinical trial safety reporting diverge in their legislative basis but converge on the standards derived from International Council for Harmonisation E2A and E6(R2) (International Council for Harmonisation, 1994; International Council for Harmonisation, 2016b). All five authorities require the sponsor to report suspected unexpected serious adverse reactions on an expedited basis. A fatal or life-threatening suspected unexpected serious adverse reaction must be reported within seven calendar days of the sponsor’s first knowledge, with a complete follow-up report required within 15 calendar days of initial notification. All other suspected unexpected serious adverse reactions must be reported within 15 calendar days.

In the European Union, clinical trial oversight was restructured by the Clinical Trials Regulation (EU) No 536/2014, fully applicable since January 2023 (European Parliament and Council, 2014). Under this regulation, sponsors submit suspected unexpected serious adverse reactions to EudraVigilance (clinical trials module), which distributes them to all concerned Member States.

In the United States, the 2010 amendments to 21 Code of Federal Regulations 312.32 revised the Investigational New Drug safety reporting framework by introducing aggregate analysis obligations (U.S. Government Publishing Office, 2023b). The December 2025 final guidance on sponsor responsibilities further consolidated this approach, clarifying that sponsors must conduct ongoing aggregate assessments of serious adverse events across the entire development program, evaluating individual cases in the context of cumulative data rather than in isolation (U.S. Food and Drug Administration, 2025a). A reporting decision for an anticipated event in the study population requires aggregate analysis, and reference safety information listed in the Investigator Brochure—which forms the basis for expectedness assessment of all suspected adverse reactions—requires aggregate assessments grounded in sponsor causality determination. Furthermore, consistent with International Council for Harmonisation E2A, a clinically important increase in the rate of an expected serious adverse drug reaction is subject to expedited reporting, which inherently requires aggregate analysis of accumulating data (International Council for Harmonisation, 1994; U.S. Food and Drug Administration, 2025a). The guidance emphasizes that safety signals often emerge from patterns identified through such analyses—for example, a series of individually unremarkable hepatic enzyme elevations that collectively may indicate dose-dependent hepatotoxicity, or frequency changes in known adverse reactions that emerge only when examined across the entire program. How to conduct such aggregate safety assessments in ongoing blinded studies without compromising study integrity remains an operational challenge not fully addressed in current International Council for Harmonisation guidance. This approach positions the annual Development Safety Update Report as the primary vehicle for comprehensive safety evaluation during clinical development, complementing expedited individual case reports that address acute safety concerns.

Japan’s Pharmaceutical and Medical Device Act imposes broadly parallel obligations; foreign suspected unexpected serious adverse reactions are reportable when they involve the same investigational compound under a Japanese Clinical Trial Notification, even if the foreign trial is conducted entirely outside Japan (Ministry of Health et al., 2021a). China similarly requires foreign suspected unexpected serious adverse reactions for compounds under Chinese Investigational New Drug to be reported to the National Medical Products Administration’s Centre for Drug Evaluation, with the same seven/15-day timelines as domestic cases (National Medical Products Administration, 2022).

### Post-marketing adverse drug reaction reporting

Post-marketing pharmacovigilance is where the most operationally significant jurisdictional differences emerge. The universal 15-calendar-day expedited reporting requirement for serious unexpected individual case safety reports is the most harmonized element of post-marketing surveillance. China’s National Medical Products Administration stands apart in requiring preliminary notification within 24 h for any post-marketing death potentially attributable to a medicinal product, followed by a complete individual case safety report within 15 calendar days (National Medical Products Administration, 2021). This reflects a policy choice to prioritize immediacy of notification over completeness of initial data.

A notable difference concerns non-serious cases. Under the European Medicines Agency’s Good Pharmacovigilance Practices Module VI (Revision 2), marketing authorization holders must submit non-serious individual case safety reports from the European Economic Area to EudraVigilance within 90 calendar days (European Medicines Agency, 2023). Health Canada, the Food and Drug Administration, the Pharmaceuticals and Medical Devices Agency, and the National Medical Products Administration do not impose individual-case reporting obligations for non-serious events; these cases are captured in aggregate periodic reports (Periodic Safety Update Reports/Periodic Benefit-Risk Evaluation Reports or Periodic Adverse Drug Experience Reports in the United States).

Japan’s re-examination system creates a particular dynamic in post-marketing reporting. During the re-examination period, marketing authorization holders under Good Post-Marketing Study Practice obligations collect serious adverse drug reactions more intensively and compile them into a re-examination report submitted to the Pharmaceuticals and Medical Devices Agency at the end of the period (Ministry of Health et al., 2021c). The 30-calendar-day timeline for serious but expected domestic adverse drug reactions is specific to Japan and has no direct counterpart in the other four systems.

In the European Union, marketing authorization holders submit serious individual case safety reports—both expected and unexpected—from the European Economic Area and qualifying third-country cases to EudraVigilance within 15 calendar days (European Medicines Agency, 2023). In Canada, serious expected and unexpected domestic adverse drug reactions require 15-day reporting (Government of Canada, 2023b). In the United States, serious unexpected individual case safety reports are reportable within 15 calendar days, while serious expected post-marketing cases are included in periodic reports rather than expedited 15-day submissions (U.S. Government Publishing Office, 2023a).

Periodic safety reporting, including Periodic Safety Update Reports and their successor Periodic Benefit-Risk Evaluation Reports (per International Council for Harmonisation E2C(R2)), is required by all five authorities (International Council for Harmonisation, 2012). During clinical development, the Development Safety Update Report (per International Council for Harmonisation E2F) serves as the comprehensive annual safety assessment (International Council for Harmonisation, 2010). The Food and Drug Administration’s proposed rule to adopt the Development Safety Update Report as the mandatory annual report format under 21 Code of Federal Regulations 312.33 would align the United States with the other four authorities, all of which already accept or require the Development Safety Update Report format (U.S. Food and Drug Administration, 2022). This convergence underscores the recognized value of structured aggregate assessments in contextualizing individual case reports within the broader safety profile.

### Summary of key differences and similarities

Table 1 presents a comparative summary of the most operationally significant reporting parameters across the five authorities.

### Medical device adverse event reporting

Medical device vigilance operates under distinct legislative frameworks from medicinal products in all five jurisdictions, though the underlying principles of seriousness-based triage and expedited reporting for deaths and serious injuries are shared. Under the European Medicines Agency’s Medical Device Regulation 2017/745 (Article 87), manufacturers must notify the relevant national competent authority within two calendar days of a serious public health threat, within 10 calendar days for incidents that led to death or unanticipated serious deterioration in health, and within 15 calendar days for other serious incidents (European Parliament and Council, 2017). The National Medical Products Administration requires 24-h notification for device-related deaths, consistent with its medicinal product framework (National Medical Products Administration, 2021). In Canada, manufacturers and importers must report device incidents that have led to death within 10 calendar days (Government of Canada, 2023c). In Japan, deaths and other serious incidents are generally reportable within 15 calendar days to the Pharmaceuticals and Medical Devices Agency/Ministry of Health, Labour and Welfare (Pharmaceuticals and Medical Devices Agency, 2022).

In the United States, device manufacturers must submit Medical Device Reports within 30 calendar days for deaths, serious injuries, and malfunctions that would be likely to cause or contribute to a death or serious injury if they were to recur (U.S. Government Publishing Office, 2023e). A five-working-day report is required only when remedial action is necessary to prevent an unreasonable risk of substantial harm to the public or when specifically requested by the Food and Drug Administration. User facilities in the United States have separate 10-working-day obligations to report deaths to both the Food and Drug Administration and the manufacturer (U.S. Government Publishing Office, 2023e).

### Vaccines and adverse events following immunization

Vaccine safety monitoring has particular public health dimensions. The immunized population is predominantly healthy, the benefit is prophylactic rather than therapeutic, and program-level coverage means that even low-frequency serious adverse events following immunization carry significant absolute numbers. In Canada, vaccine safety operates through a dual-track system: marketing authorization holders report to Health Canada’s Canada Vigilance Programme, while healthcare providers and public health officials report to the Public Health Agency of Canada through the Canadian Adverse Events Following Immunization Surveillance System (Health Canada, 2019). Japan similarly employs dual reporting (Pharmaceuticals and Medical Devices Agency, 2022). The United States employs the Vaccine Adverse Event Reporting System, jointly managed by the Food and Drug Administration and Centers for Disease Control and Prevention (U.S. Food and Drug Administration, 2012). China’s vaccine-specific framework is reinforced by the Vaccine Administration Law (2019), with 24-h reporting for vaccine deaths (National People’s Congress, 2019b). Cluster or lot-specific adverse events following immunization trigger immediate emergency protocols across all five jurisdictions.

### Special populations: pregnancy and pediatric adverse events

Pregnancy exposure and pediatric adverse events are designated as special populations in Good Pharmacovigilance Practices Module VI, International Council for Harmonisation E2C(R2), and equivalent guidance from all five authorities (International Council for Harmonisation, 2012; European Medicines Agency, 2023). Congenital anomalies and fetal or neonatal death following *in-utero* drug exposure meet International Council for Harmonisation E2A seriousness criteria by definition and therefore require expedited individual case safety report reporting within 15 calendar days (International Council for Harmonisation, 1994). The National Medical Products Administration requires 24-h preliminary notification for neonatal deaths following pregnancy exposure, consistent with its general post-marketing death-reporting framework (National Medical Products Administration, 2021). Pediatric adverse events carry particular significance given the historical under-representation of children in clinical trials and the consequent prevalence of off-label pediatric prescribing.

### Special situations: lack of efficacy, medication errors, and abuse

#### Lack of efficacy

The reportability of lack of efficacy is nuanced. International Council for Harmonisation E2D and Good Pharmacovigilance Practices Module VI clarify that lack of efficacy alone does not constitute an adverse drug reaction unless it leads to a medically significant clinical outcome (International Council for Harmonisation, 2003; European Medicines Agency, 2023). Lack of efficacy becomes reportable when its clinical consequence meets the seriousness threshold, most typically with products whose therapeutic failure carries life-threatening implications, including vaccines, anticoagulants, antiepileptics, antiretrovirals, and insulin. Health Canada’s C.01.017(2)(b) creates a specific obligation to report, within 15 calendar days, any unusual failure of a drug to produce its intended effect for domestic cases, independent of International Council for Harmonisation seriousness criteria (Government of Canada, 2023b). In practice, this provision is intended to capture life-sustaining therapy failures and unexpected efficacy signals suggesting quality or supply issues. Whether expedited reporting of individual lack-of-efficacy cases has in practice revealed antimicrobial resistance patterns earlier than routine surveillance is not well documented in the peer-reviewed literature, and this remains an area where empirical evaluation would strengthen the rationale for such provisions.

#### Medication errors

Medication errors are reportable to all five authorities when they result in an adverse event, regardless of whether the resulting harm was expected or unexpected. The underlying principle is that the product characteristics (labeling, packaging, naming) may have contributed to the error, making it a product safety issue even when causality is indirect. All authorities expect medication errors to be documented in individual case safety report narratives with sufficient detail to permit root-cause analysis; the European Medicines Agency’s Good Pharmacovigilance Practices Module VI provides structured guidance on error classification through coding conventions (European Medicines Agency, 2023).

#### Abuse, misuse, and occupational exposure

Adverse events arising from abuse, misuse, off-label use, or occupational exposure are subject to standard seriousness-based triage in all five systems. For controlled substances, the Pharmaceuticals and Medical Devices Agency and the National Medical Products Administration require parallel notification to narcotics control bodies in addition to standard pharmacovigilance reporting (Pharmaceuticals and Medical Devices Agency, 2022; National Medical Products Administration, 2022). The Food and Drug Administration’s Risk Evaluation and Mitigation Strategy program creates enhanced monitoring obligations for drugs with abuse potential or other serious safety concerns (U.S. Food and Drug Administration, 2012).

## Discussion

### Jurisdictional distinctions and their implications

The comparative analysis reveals a pharmacovigilance landscape that is harmonized at the level of core definitions (seriousness, expectedness, and minimum case validity) but divergent in operational implementation, including timelines, submission systems, language requirements, and scope of reportable events.

China’s 24-h post-marketing death notification is the most stringent individual-case death-reporting requirement among the five authorities and creates a dual-speed workflow: immediate preliminary notification followed by a complete individual case safety report within 15 days (National Medical Products Administration, 2021). While this reflects strong regulatory commitment to rapid awareness of fatal outcomes, the operational challenges merit consideration. For global marketing authorization holders managing cases across multiple time zones with translation and medical review requirements, the 24-h timeline frequently necessitates submission of incomplete preliminary data. The incremental safety value of preliminary notification at 24 h compared to a single complete report at 15 days—the timeline used by the other four authorities—remains an area for empirical evaluation.

The European Medicines Agency’s 90-calendar-day submission requirement for non-serious European Economic Area individual case safety reports is a distinctive feature in global pharmacovigilance (European Medicines Agency, 2023). The theoretical rationale for this provision is that patterns in non-serious events may precede recognition of serious outcomes, and the 90-day timeline provides sufficient time for case validation without compromising signal detection timeliness. However, it should be acknowledged that this rationale, while scientifically plausible, rests primarily on regulatory judgment rather than robust empirical evidence demonstrating that individual-case submission of non-serious reports has yielded safety signals earlier than aggregate periodic assessments. From an operational standpoint, the volume of non-serious individual case safety reports submitted under this provision creates a substantial data accumulation in EudraVigilance. Whether this volume of data is more productively analyzed through individual case submission and database mining, or through structured aggregate review in annual Periodic Benefit-Risk Evaluation Reports, is a question that merits further study. Regulatory experience suggest that aggregate assessments in the Development Safety Update Report or Periodic Benefit-Risk Evaluation Report may be better suited to detecting the patterns in non-serious events that give rise to meaningful safety signals, precisely because they provide the contextual framework—exposure data, temporal trends, subgroup analyses—necessary for interpretation (International Council for Harmonisation, 2012; International Council for Harmonisation, 2010).

Japan’s re-examination system imposes enhanced post-marketing surveillance obligations that yield high-quality domestic safety data during the critical four-to-ten-year period following approval (Ministry of Health et al., 2021c). The 30-day timeline for serious expected adverse drug reactions, unique among the five authorities, enables earlier detection of frequency changes in known risks. While this creates additional reporting burden without international precedent, the approach has proven valuable for identifying emerging safety signals during the period of greatest uncertainty about a product’s safety profile.

Health Canada’s C.01.017(2)(b) provision requiring 15-day reporting of unusual lack of efficacy for domestic cases addresses a gap in conventional pharmacovigilance frameworks (Government of Canada, 2023b). Therapeutic failures in life-sustaining treatments—anticoagulants, antiepileptics, antiretrovirals, insulin, and antimicrobials—may signal product quality issues or incorrect dosing guidance even when no traditional adverse reaction occurs. However, whether this provision has led to earlier detection of specific safety signals—such as antimicrobial resistance patterns—through expedited individual case reporting rather than through microbiological surveillance or aggregate periodic reports has not been well documented. This represents a hypothesis with strong clinical logic, but published evidence supporting its practical impact on signal detection is limited.

### The role of aggregate analysis: DSUR and PBRER as complementary tools

A central theme emerging from this comparative analysis is the growing recognition that effective pharmacovigilance depends on the synergy between expedited individual case reporting and structured aggregate assessments. The Food and Drug Administration’s 2010 amendments to 21 Code of Federal Regulations 312.32 introduced aggregate analysis obligations for Investigational New Drug safety reporting, and the December 2025 final guidance on sponsor responsibilities further consolidated this approach (U.S. Government Publishing Office, 2023b; U.S. Food and Drug Administration, 2025a). The Food and Drug Administration’s proposed transition from the Investigational New Drug Annual Report to the Development Safety Update Report format represents a significant step toward global harmonization of periodic aggregate assessment (U.S. Food and Drug Administration, 2022).

The Development Safety Update Report (International Council for Harmonisation E2F) during clinical development and the Periodic Benefit-Risk Evaluation Report (International Council for Harmonisation E2C(R2)) during the post-marketing phase provide structured vehicles for comprehensive safety evaluation that complement expedited individual case reporting (International Council for Harmonisation, 2012; International Council for Harmonisation, 2010). These aggregate reports enable identification of patterns that individual case reports alone may not reveal: dose-response relationships, subpopulation-specific risks, frequency changes in known adverse reactions, and emerging safety signals that manifest as subtle deviations from expected reporting patterns. The annual periodicity of the Development Safety Update Report, combined with its requirement for a comprehensive benefit-risk assessment, provides a structured opportunity for medical safety review that contextualizes the sometimes overwhelming volume of individual case reports accumulated over the reporting interval.

This perspective has implications for the debate regarding non-serious case reporting. While the European Medicines Agency’s requirement for 90-day submission of individual non-serious cases from the European Economic Area ensures that these data enter EudraVigilance for potential signal detection, the other four authorities rely on aggregate periodic reports to capture non-serious event data. Both approaches have merits: individual case submission provides granular data that can be queried and mined, while aggregate assessment in Periodic Benefit-Risk Evaluation Reports provides contextual interpretation. The optimal approach may depend on the maturity of the pharmacovigilance database infrastructure, the sophistication of signal detection algorithms, and the resources available for data quality assurance.

### Integrated assessment of pharmacovigilance frameworks

No single authority demonstrates superiority across all dimensions. The European Medicines Agency achieves broad data collection through systematic non-serious case reporting and centralized infrastructure, while maintaining operationally realistic timelines. The Food and Drug Administration demonstrates practical risk-based prioritization, focusing expedited reporting on unexpected signals while capturing expected events in periodic reports, and has led in emphasizing aggregate analysis—most recently through the December 2025 final guidance and the proposed Development Safety Update Report rule. Health Canada’s lack of efficacy provision and Japan’s re-examination system represent valuable jurisdiction-specific innovations. The National Medical Products Administration’s 24-h death notification, while operationally challenging, reflects strong regulatory commitment to immediate awareness of fatal outcomes.

The optimal pharmacovigilance framework would integrate the strengths of multiple systems: comprehensive data collection with operationally realistic timelines; aggregate analysis emphasis through Development Safety Update Reports and Periodic Benefit-Risk Evaluation Reports; special situation provisions for lack of efficacy and other non-standard signals; intensive early post-marketing surveillance; and timeline stratification that balances urgency with data quality. Critically, such a framework would recognize that the most informative safety oversight arises from the interplay between medical safety review of individual cases, medical monitoring of individual studies, and aggregate assessment of the cumulative safety profile—rather than from maximizing the volume or speed of individual case submissions alone.

### Device vigilance: divergent approaches to urgency

In device vigilance, the European Union’s Medical Device Regulation establishes a differentiated set of timelines that appropriately stratify urgency: 2 days for serious public health threats, 10 days for deaths or unanticipated serious deterioration in health, and 15 days for other serious incidents (European Parliament and Council, 2017). This risk-based approach balances the need for rapid public health action in urgent situations with realistic timelines for thorough investigation of complex device incidents. The Food and Drug Administration’s default 30-calendar-day manufacturer Medical Device Report timeline, while more operationally realistic for device investigations that often require technical analysis and root-cause determination, may delay regulatory response in situations requiring immediate action (U.S. Government Publishing Office, 2023e). Global device manufacturers often operationalize the most stringent applicable timelines to ensure cross-jurisdictional compliance.

### Infrastruc ture and statistical power

Centralized databases with sophisticated analytics capabilities are essential for modern signal detection. For example, Canada Vigilance Program and the FDA Adverse Event Reporting System both provide public access to anonymized individual case safety report data, supporting independent signal detection by researchers, regulators, and the pharmaceutical industry (U.S. Food and Drug Administration, 2012; Health Canada, 2022). Population size significantly affects statistical power for rare event detection. China’s 1.4 billion population and the European Union’s 450 million provide substantial advantages over Canada’s 38 million for detecting low-frequency signals. However, signal detection depends not only on absolute case numbers but also on reporting rates, data quality, and analytical sophistication. Mature systems with lower absolute volumes but higher reporting rates and data quality may outperform larger systems with developing infrastructure (Pal et al., 2013).

### Emerging trends and future directions

Emerging trends include expanding use of artificial intelligence and machine learning tools for signal detection in large spontaneous reporting databases. Natural language processing can extract structured information from narrative fields, enabling more sophisticated pattern recognition (Koutkias and Jaulent, 2015). Predictive algorithms can identify potential signals earlier by detecting subtle deviations from expected reporting patterns. However, these advanced analytics require high-quality, standardized data—reinforcing the importance of International Council for Harmonisation E2B(R3) adoption and consistent data collection practices (International Council for Harmonisation, 2016a).

The progressive implementation of International Council for Harmonisation E2B(R3) across all five authorities is reducing formatting and transmission burdens, enabling marketing authorization holders to focus resources on substantive safety science rather than administrative compliance. The convergence toward the Development Safety Update Report as the global standard for annual safety assessment during clinical development is a particularly significant harmonization achievement. However, jurisdiction-specific differences in timelines, language requirements, destination systems, and special-situation provisions will persist, requiring continued investment in global pharmacovigilance infrastructure and expertise.

### Limitations

This comparative analysis has several limitations. First, as a narrative review rather than systematic review, our literature search may not have captured all relevant regulatory guidance documents or peer-reviewed literature, particularly recent updates or jurisdiction-specific interpretational guidance. Second, the analysis focuses on formal regulatory requirements as stated in legislation and guidance documents, which may not fully reflect actual implementation practices or enforcement priorities in each jurisdiction. Third, we did not conduct empirical research on the operational burden or signal detection effectiveness of different reporting frameworks, and several of our assessments—particularly regarding the value of non-serious case reporting and the role of aggregate assessments—represent the authors’ informed professional judgments rather than empirically validated conclusions. Fourth, the rapidly evolving nature of pharmacovigilance regulation, particularly in China and with respect to the Food and Drug Administration’s proposed Development Safety Update Report rulemaking, means that some requirements may change between manuscript preparation and publication. Fifth, additional regulatory authorities not included in this analysis—including the United Kingdom Medicines and Healthcare products Regulatory Agency, the Therapeutic Goods Administration of Australia, and India’s Pharmacovigilance Programme—would broaden the comparative perspective and should be addressed in future research. Finally, our qualitative assessments of “completeness” and “realism” involve subjective judgment informed by regulatory expertise but not validated through formal stakeholder consultation or empirical testing.

## Conclusion

Global pharmacovigilance compliance requires marketing authorization holders and sponsors to maintain fluency with five distinct regulatory frameworks while leveraging the harmonized International Council for Harmonisation foundation. The decision algorithms described in this analysis (available as Supplementary Material) provide a practical tool for triage from case receipt to reporting obligation determination, highlighting where authority-specific requirements diverge from the International Council for Harmonisation baseline.

The comparative analysis reveals that no single authority demonstrates superiority across all dimensions of safety signal collection. Each framework reflects different policy priorities and operational trade-offs: the European Medicines Agency’s broad data collection scope, the Food and Drug Administration’s aggregate analysis emphasis, Health Canada’s lack of efficacy provision, Japan’s intensive early post-marketing surveillance, and the National Medical Products Administration’s 24-h death notification each address distinct aspects of the pharmacovigilance challenge.

A central conclusion of this analysis is that effective pharmacovigilance depends not on any single reporting mechanism but on the complementary interplay between expedited individual case reporting, medical monitoring of individual studies, and structured aggregate assessments through Development Safety Update Reports and Periodic Benefit-Risk Evaluation Reports. The Food and Drug Administration’s December 2025 final guidance and proposed Development Safety Update Report rule, the European Medicines Agency’s mature Good Pharmacovigilance Practices framework, and the global adoption of International Council for Harmonisation E2F all reflect convergence toward this integrated approach.

Future harmonization efforts should prioritize evidence-based evaluation of reporting requirements—including whether individual case submission of non-serious events yields superior signal detection compared to aggregate periodic assessment—and continue the progress toward globally standardized aggregate report formats. Pharmacovigilance professionals are best served by structured decision frameworks, global infrastructure capable of meeting the most stringent applicable timelines, and ongoing engagement with evolving regulatory expectations across all five major authorities. The ultimate measure of pharmacovigilance system effectiveness is not the volume of reports submitted or the speed of submission, but the ability to detect safety signals early, assess them rigorously through both individual case review and aggregate analysis, and communicate risks effectively to protect patient safety.

## Data Availability

The original contributions presented in the study are included in the article/Supplementary Material, further inquiries can be directed to the corresponding author.
