# Supplementary material for: Global pharmacovigilance reporting: comparative analysis of adverse event obligations across five major regulatory authorities
Source: Front Drug Saf Regul. 2026 May 7;6:1821686. doi: 10.3389/fdsfr.2026.1821686 (PMC13189745; doi:10.3389/fdsfr.2026.1821686)
Supplement: Supplementary file 1 [file DataSheet1.pdf]

Supplementary Figure S1. Integrated Decision Algorithm for Global Adverse Event Reporting Obligations

Page 1 of 2: Case Receipt to Authority-Specific Reporting Pathway

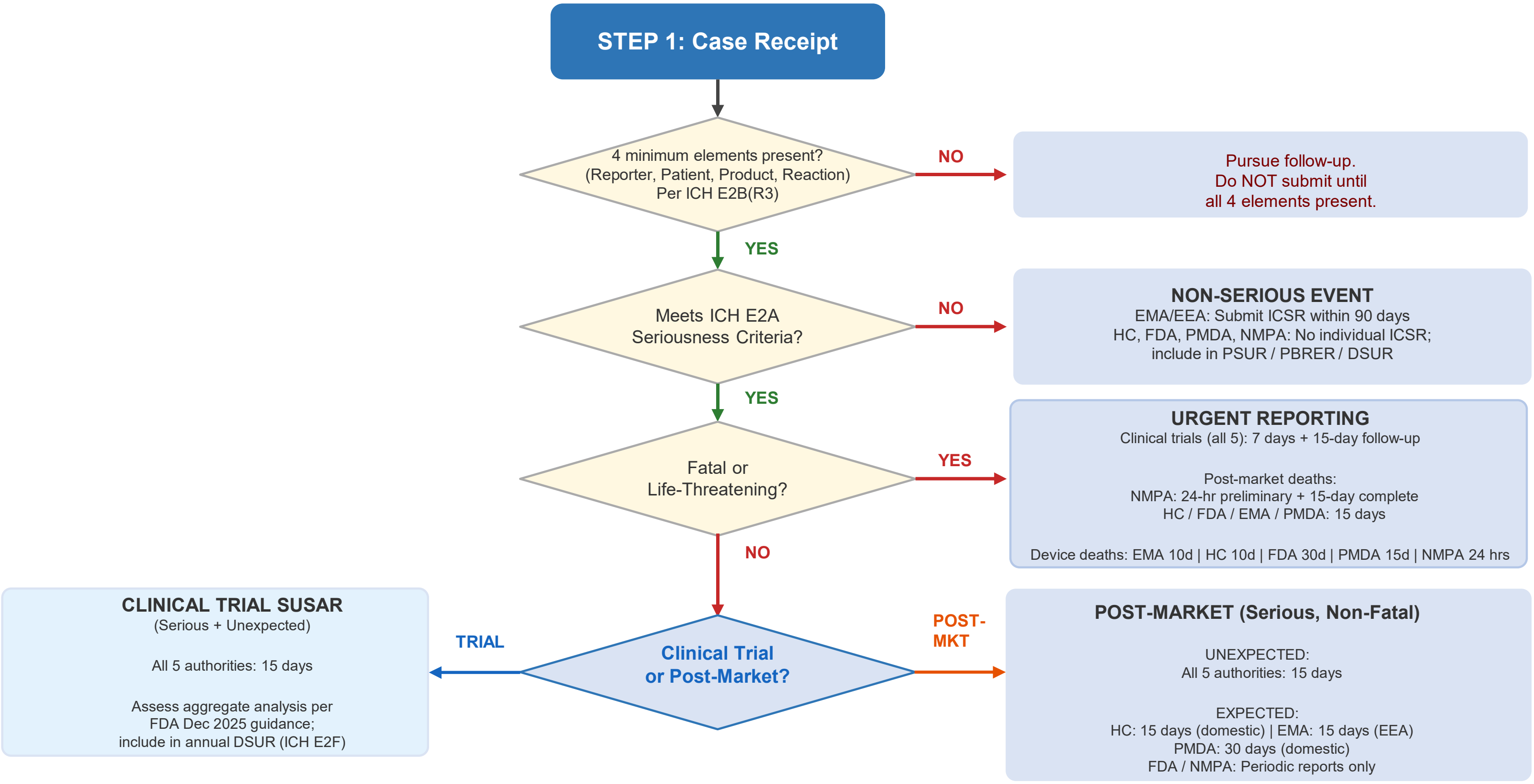

DSUR, Development Safety Update Report; EEA, European Economic Area; HC, Health Canada; ICH, International Council for Harmonisation; ICSR, Individual Case Safety Report; NMPA, National Medical Products Administration; PBRER, Periodic Benefit-Risk Evaluation Report; PMDA, Pharmaceuticals and Medical Devices Agency; SUSAR, Suspected Unexpected Serious Adverse Reaction.

Supplementary Figure S1 (continued)

Page 2 of 2: Special Situations, Device Vigilance, and Aggregate Reporting

A. Special Situations Decision Path

Lack of Efficacy

Does failure meet seriousness criteria?
- YES → Report as serious ADR (15 days)
- NO + Canadian product → HC C.01.017(2)(b): Report unusual failure within 15 days
- NO + Other jurisdictions → Periodic reports only
- NMPA: Emphasis on antimicrobial LOE

Medication Errors

Did error result in adverse event?
- YES → Report per seriousness-based triage
- Document root-cause in ICSR narrative
- EMA GVP Module VI: structured coding

Pregnancy Exposure

Congenital anomaly or fetal/neonatal death?
- YES → 15 days (all); NMPA: 24 hrs if death
- Normal outcome → Document in periodic reports

Key Principle: Complementary Safety Architecture

Effective pharmacovigilance depends on the interplay between:
1. Medical safety review of individual cases (expedited ICSRs)
2. Medical monitoring of individual studies (ongoing oversight)
3. Aggregate assessment of cumulative safety profile (DSUR / PBRER)

Focus on informative events; leverage aggregate reports to contextualize volume.

B. Device Vigilance Comparative Timelines

Event Type	EMA	HC	FDA	PMDA	NMPA
Serious public health threat	2 days	—	—	—	—
Death / serious deterioration	10 days	10 days	30 days	15 days	24 hours
Other serious incidents	15 days	Report	30 days	15 days	15 days
Remedial action (US only)	—	—	5 work d	—	—

C. Aggregate Reporting: DSUR & PBRER Framework

CLINICAL DEVELOPMENT (DSUR — ICH E2F)

Annual comprehensive safety assessment
All 5 authorities accept/require DSUR format
FDA proposed rule: replace IND Annual Report with DSUR
Integrates: cumulative exposure, interval & cumulative safety data,
known risks, benefit-risk assessment
Submit within 60 days of data lock point

POST-MARKETING (PBRER — ICH E2C(R2))

Periodic benefit-risk evaluation
EMA: EU Reference Dates list sets schedule
FDA: PADER quarterly/annual per 21 CFR 314.81
HC / PMDA / NMPA: Product-specific schedules
Captures non-serious events from all jurisdictions

ADR, Adverse Drug Reaction; DSUR, Development Safety Update Report; GVP, Good Pharmacovigilance Practices; LOE, Lack of Efficacy; PADER, Periodic Adverse Drug Experience Report; PBRER, Periodic Benefit-Risk Evaluation Report.
